# Spiny Mice Show a Profibrotic Epicardial Mesothelial Response to Hypoxic Injury Comparable to C57BL/6 Mice

**DOI:** 10.3390/biom16050717

**Published:** 2026-05-13

**Authors:** Konstantin Dergilev, Aleria Dolgodvorova, Zoya Tsokolaeva, Irina Iarushkina, Irina Beloglazova, Yulia Goltseva, Yelena Parfyonova

**Affiliations:** 1 Institute of Experimental Cardiology Named after Academician V.N. Smirnov, Federal State Budgetary Institution National Medical Research Center of Cardiology Named after Academician E.I. Chazov, Ministry of Health of the Russian Federation, 121552 Moscow, Russia; 2 Federal Research and Clinical Center of Intensive Care Medicine and Rehabilitology, 141534 Moscow, Russia

**Keywords:** mesothelial cells, hypoxia, *Acomys cahirinus*, myocardial infarction, epicardium, cardiac repair

## Abstract

Epicardial mesothelium plays a pivotal role in postinfarction cardiac repair by generating fibroblasts, producing extracellular matrix, and releasing paracrine mechanisms. However, interspecies differences have not been sufficiently studied, particularly in in vivo models of scar-free healing such as the African spiny mouse (*Acomys cahirinus*). This study aimed to compare the profibrotic response of epicardial mesothelial cells (MCs) from *Acomys* and C57BL/6 mice to hypoxic stress, a key factor in postinfarction recovery. We isolated epicardial MCs from the African spiny mouse (*Acomys cahirinus*), a species with documented cardiac regenerative capabilities, and from C57BL/6 laboratory mice. Using a CoCl_2_-induced hypoxia model in vitro, we assessed cell viability, morphological changes, and expression of epithelial and fibroblast markers. In vivo, following experimental myocardial infarction (MI), we evaluated tissue hypoxia (pimonidazole adducts), epicardial activation (layer thickness, Wt1^+^ and TBX18^+^ progenitor cells), and collagen accumulation. The study was conducted using real-time PCR, Western blotting, immunohistochemical analysis and microscopic examination. In vitro, MCs from both species exhibited an epithelial-like phenotype under normoxic conditions, expressing E-cadherin and cytokeratin 18. Hypoxia (200 µM CoCl_2_) induced a comparable response in both *Acomys* and C57BL/6 cells, characterized by a shift to a spindle-shaped, fibroblast-like morphology, decreased E-cadherin expression, and increased pro-collagen 1 and α-SMA expression. Following MI, both species exhibited similarly extensive hypoxic areas affecting the epicardial zone. Epicardial activation dynamics were comparable: from day 3 post-MI, epicardial thickness increased significantly, and Wt1^+^ and TBX18^+^ progenitor cells accumulated, peaking during the first week. Collagen accumulation in the epicardial region was similar between species, although the number of Wt1^+^ cells was higher in C57BL/6 on day 7. Despite the well-known superior regenerative capacity of spiny mice, epicardial MCs from *Acomys* and C57BL/6 demonstrated similar signs of profibrotic responses to hypoxic stimulation both in vitro and following MI. These findings suggest that species-specific regenerative outcomes may not be attributable to differential acute epicardial sensitivity to hypoxia, but rather to downstream mechanisms or additional factors influencing the cardiac repair process. This study provides the first characterization of *Acomys* epicardial MCs and establishes a foundation for further investigation of evolutionarily conserved and species-specific mechanisms of cardiac regeneration.

## 1. Introduction

Cardiovascular diseases continue to be the leading cause of mortality worldwide, with myocardial infarction (MI) being one of the most severe disorders [1,2]. The high morbidity and mortality associated with MI can be attributed to the limited reparative capacity of the adult heart, leading to scar tissue formation, pathological remodeling, and the eventual development of heart failure [3,4]. Therefore, understanding the cellular and molecular mechanisms initiated by hypoxia that govern postinfarction fibrosis is imperative for developing effective therapeutic strategies aimed at mitigating adverse remodeling and reducing the progression from MI to heart failure [5].

In this context, researchers have increasingly focused on the cellular and molecular drivers of fibrosis-associated endogenous repair mechanisms observed across different animal species. One of the key players is the epicardium, the visceral layer of the pericardium, which forms a thin membranous layer covering the heart and the roots of the great vessels [6]. The epicardium is composed of MCs located on a basement membrane, in close proximity to the extracellular matrix (ECM), lymphatic and blood vessels, fibroblasts, and cardiomyocytes. This strategic position allows the epicardium to create a specialized microenvironment that acts as a regulatory hub controlling reparative processes in the heart [7]. In the developing heart, it serves as the primary source of fibroblasts [8,9], contributes to the formation of various cardiac cell types [10,11], and plays a role in the construction of the coronary vascular network [12,13]. Furthermore, the epicardium modulates intercellular communication by producing ECM components [14,15] and paracrine factors [15,16,17].

In the adult heart, the epicardial MCs can be reactivated by hypoxia, triggering an embryonic repair program that leads to the formation of Wt1^+^, Tbx18^+^, and Tcf21^+^ epicardial-derived progenitor cells [18]. These cells contribute to a pool of fibroblasts and are involved in scar formation and repair [18,19,20,21,22]. Despite these findings, the specific molecular pathways through which hypoxia influences MCs’ behavior remain unclear, particularly the mechanisms of fibroblast differentiation, interspecies differences, and the temporal dynamics of epicardial activation [23]. Although hypoxia-inducible factors, HIF-1α and HIF-2α, are recognized as central regulators of cellular responses to low oxygen levels, their precise role in epicardial epithelial–mesenchymal transition (EMT), fibroblast-like differentiation, and paracrine signaling varies between species and developmental stages [24,25,26].

Consequently, the fundamental role of the reactivated epicardium in cardiac repair has been demonstrated in zebrafish and salamanders [27,28]. This includes modulation of inflammatory responses and ECM composition, secretion of paracrine factors, and supply of cells to the damaged heart. However, the behavior of epicardial MCs in mammals in response to damaging stimuli remains largely unexplored. This knowledge gap has prompted us to focus our research on the epicardium mesothelium of the African spiny mouse (*Acomys cahirinus)*, a mammal with remarkable abilities to regenerate various tissues with minimal fibrosis [29,30]. Furthermore, *Acomys* exhibits substantial cardiac regeneration and repair following myocardial infarction, evidenced by a high tolerance to ischemia, reduced scarring, augmented blood vessel formation, and functional recovery [31,32,33]. Given that *Acomys cahirinus* is phylogenetically closer to humans than traditional model organisms such as zebrafish or newts, the study of evolutionarily conserved mechanisms controlling fibrosis and cardiac repair in this species is of particular interest.

The present study aims to assess the profibrotic response of epicardial MCs from *Acomys cahirinus* to hypoxic stress as a key factor in the post-infarction recovery process in comparison with C57Bl/6 laboratory mice.

## 2. Materials and Methods

### 2.1. Animals

The work was performed on male C57BL/6 mice (16- to 18-week-old) and male *Acomys cahirinus* (18- to 20 weeks old). These mice were housed in standard polypropylene cages under controlled vivarium conditions (temperature 20–24 °C, humidity 35–65%, 12 h light/dark cycle).

Animal care and handling followed the guidelines outlined in the European Convention for the Protection of Vertebrate Animals Used for Experimental and Other Scientific Purposes (ETS No. 123). All procedures were conducted in compliance with Directive 2010/63/EU of the European Parliament and of the Council of 22 September 2010 regarding the protection of animals used for scientific purposes. The study protocols received approval from the local ethics committee of the National Medical Research Center of Cardiology, named after Academician E. I. Chazov (permit #LA/20.12.24 (29 January 2025)).

### 2.2. Myocardial Infarction Modeling

MI was induced in adult C57BL/6 mice and spiny mice following the established protocol described previously [34]. In brief, the animals were anesthetized with Tribromoethanol (Avertin) 100 mg/kg body weight, injected intraperitoneally, intubated, and positioned on a MiniVent mechanical ventilator (Hugo Sachs Elektronik Harvard Apparatus GmbH, March, Germany). To induce MI, the proximal segment of the left coronary artery was surgically ligated via an incision in the ninth left intercostal space. In the sham-operated group, the heart was exposed through a thoracotomy, but the coronary artery was left intact without placing a ligature.

### 2.3. Pimonidazole-Based In Vivo Hypoxia Analysis

Hypoxia in cardiac tissue was assessed using the Hypoxyprobe Kit (Catalog No. HPK-2025, Biotracker Inc., Mountain View, CA, USA), a pimonidazole-based method that relies on the formation of covalent protein adducts in viable hypoxic cells. Staining for pimonidazole bound to hypoxic zones in vital tissues during hypoxia was completed via IP injection of the hypoxic marker Hypoxyprobe-1 (60 mg/kg pimonidazole, Hypoxyprobe, Burlington, MA, USA) diluted in 0.9% sterile saline (total volume 100 µL) 60 min before euthanasia. The hearts were immediately excised, frozen in embedding medium (Tissue-Tek^®^ O.C.T. Compound (Sakura)) in liquid nitrogen vapor. Eight consecutive parasternal short-axis sections of the heart (7 µm thick) were prepared and then stained immunohistochemically using antibodies from the Hypoxyprobe OmniKit and ImmPRESS polymer reagents, ImmPACT DAB, and phosphatase substrates (Vector Laboratories, Newark, CA, USA) according to the manufacturer’s recommendations. Five to eight random areas per slide were analyzed for positive pimonidazole staining and analyzed using NIS-Elements NIS-Elements AR software (v 5.42.01) with the artificial intelligence module and the General Analysis 3 pipeline.

### 2.4. Immunohistochemical Analysis

Expression of the epicardial progenitor cell markers Wt1 (Abcam, Waltham, MA, USA) and TBX18 (Abcam, Waltham, MA, USA) in the hearts of *Acomys cahirinus* and C57BL/6 mice was detected by immunohistochemical staining of tissue cryosections. The staining procedure was performed using ImmPRESS polymer reagents and ImmPACT phosphatase substrate (Vector Laboratories, Newark, CA, USA) according to the manufacturer’s recommendations. The number of Wt1+ and TBX18+ nuclei was then normalized to the total epicardium area and analyzed using NIS-Elements software with the Segment.ai artificial intelligence module and the General Analysis 3 pipeline.

### 2.5. Real-Time PCR Analysis

The mRNA levels of the profibrotic genes were determined by real-time PCR in epicardial mesothelial cells obtained on day 3 after MI from the hearts of *Acomys cahirinus* and C57BL/6. Cell samples were obtained using a previously described method [35] by treating hearts with a 0.25% trypsin solution (Paneco, Moscow, Russia) 3 times for 5 min, followed by mechanical scraping from the heart surface. Total RNA was extracted from MCs using an RNeasy Mini Kit (QIAGEN, Germantown, MD, USA) and used in the synthesis of cDNA with MMLV RT Kit (Eurogene, Moscow, Russia). The real-time PCRs were performed using Syber Green^®^ intercalating dye (Eurogene, Moscow, Russia) on StepOnePlusTM Real-Time PCR System (ThermoFisher Scientific, Waltham, MA, USA). The relative mRNA expression normalized by β-actin was quantitatively calculated by the ΔΔCt method. The sequences of the primers used in real-time PCR are listed in Table 1.

### 2.6. Epicardial Collagen Content and Thickness

Epicardial thickness was measured on hematoxylin-eosin-stained cryosections using ImageJ software (v 1.54d, NIH, USA). For each cryosection, the width was measured five times in the epicardium overlying the scar area and averaged. For each animal, this was repeated on 4–5 cryosections, and the values were averaged to obtain a single value per animal. Collagen content in the intact and activated epicardium (3, 7 and 14 days after MI) was analyzed by staining heart cryosections with Picrosirius Red following a previously described protocol [36]. The distribution of collagen per unit area of the activated epicardium was quantified using NIS-Elements AR software (v 5.42.01) with the Segment.ai artificial intelligence module and the General Analysis 3 pipeline.

### 2.7. Epicardial Mesothelial Cell Isolation and Cultivation

The modified method for obtaining epicardial mesothelial cells was based on a previously described protocol [37]. Briefly, epicardial MCs were isolated by treating hearts from *Acomys cahirinus* and C57BL/6 mice with a 0.25% trypsin solution (Paneco, Moscow, Russia) at 37 °C for 20 min under gentle rotation. This procedure was repeated three times to increase the efficiency of isolation. The resulting cell suspension was centrifuged at 350× *g* for 5 min at 4 °C, the supernatant was discarded, and the cells were resuspended in RPMI-1640 medium supplemented with 10% FBS, 1% penicillin-streptomycin (culture medium), and 10 µM SB 431542. MCs were seeded onto Petri dishes pre-coated with Geltrex diluted 1:30 in culture medium. The medium was changed 3–4 h after isolation, then the following day, and every 2–3 days thereafter as needed. Early-passage cells (P1–2) were used for hypoxia-associated experiments in vitro.

### 2.8. Chemically Induced Hypoxia Modeling

Hypoxia was induced in C57BL/6 and *Acomys* mesothelial cells by adding CoCl_2_ according to a previously described protocol [37]. To analyze morphological changes and fibroblast-specific protein expression, C57BL/6 mice and *Acomys* MCs were treated with 200 µM CoCl_2_ for 72 h.

### 2.9. Cell Viability/Proliferation Assay

C57BL/6 mouse and *Acomys* MCs were plated in 96-well culture plates (2 × 10^3^ cells/well). Hypoxia was induced by adding CoCl_2_·6H_2_O to the medium at the following concentrations: 100 µM, 200 µM, 300 µM, 400 µM and 500 µM. The cells were incubated for 24 h in RPMI-1640 supplemented with 1% FBS and 1% antibiotic-antimycotic. Subsequently, 10 µL of the fluorescent dye PrestoBlue™ Cell Viability Reagent (Invitrogen, Carlsbad, CA, USA) was added to the culture medium for an additional hour. Fluorescence intensity was measured using a Victor X3 spectrophotometer (PerkinElmer, Waltham, MA, USA) at a wavelength of 570 nm, and the number of cells per well was determined by plotting a standard curve.

### 2.10. Western Blot Analysis

Western blotting was performed using specific antibodies against Collagen 1 (Abclonal, Wuhan, China), α-SMA (Abclonal, Wuhan, China), beta-Tubulin (Cell Signaling, Boston, MA, USA), E-cadherin (Abclonal, Wuhan, China), and Cytokeratin 18 (Abclonal, Wuhan, China), in accordance with a previously described protocol [38].

### 2.11. Statistical Analysis

The data are presented as mean ± SD. Unless otherwise specified, each experiment was replicated three times. The statistical significance of treatment differences was assessed using a Mann–Whitney U-test and one or two-way analysis of variance (ANOVA) with Tukey’s multiple comparisons test using GraphPad Prism software (version 8, GraphPad, La Jolla, CA, USA). A *p*-value less than 0.05 indicated statistically significant results.

## 3. Results

### 3.1. In Vitro Analysis of Acomys and C57BL/6 Epicardial Cells Revealed Mesothelial-Like Characteristics

**Figure 1:**
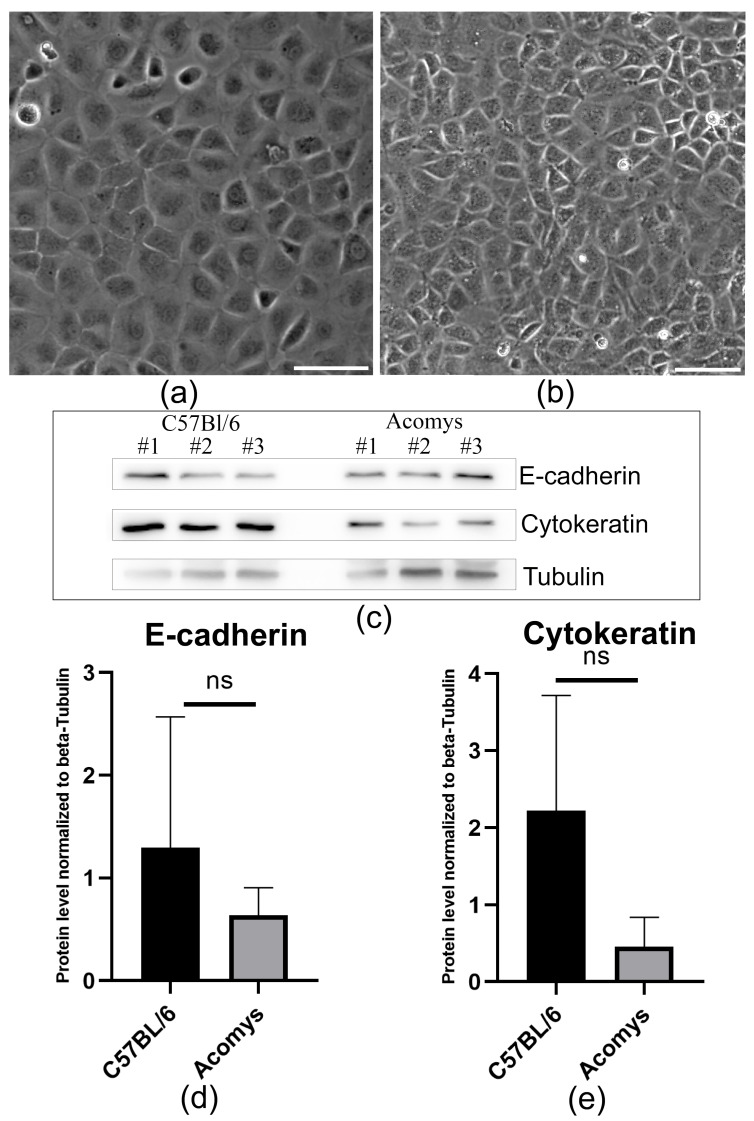
Characterization of epicardial-derived MCs. (**a**,**b**) Representative phase-contrast images showing MCs obtained from the heart of *Acomys cahirinus* (**a**) and C57BL/6 (**b**); Scale bar—100 μm; (**c**) Immunoblotting data showing the expression of E-cadherin, cytokeratin, and tubulin proteins in MCs obtained from the hearts of *Acomys cahirinus* and C57BL/6. All data were obtained from cells isolated in three independent experiments. The labels “#1, #2, #3” refer to the numbers of the independent experimental replicates (original images can be found in Figure S2); (**d**,**e**) Graphs quantifying E-cadherin (**d**) and cytokeratin (**e**) expression in MCs obtained from the hearts of *Acomys cahirinus* and C57BL/6. Statistical analysis was performed using the Mann–Whitney U test (n = 3).

MCs are a fundamental component of the epicardium [39]. Using a combination of enzymatic dissociation and mechanical manipulation, we isolated an enriched population of epicardial mesothelial cells from the hearts of *Acomys* mice and C57BL/6. Following this, a comparison was performed between these cells and their counterparts from C57BL/6 mice (*Mus musculus*). Both cell types exhibited an epithelial-like morphology (Figure 1a,b), formed a single layer of flattened, pavement-like squamous cells and expressed the specialized adherent junction component E-cadherin (Figure 1c,d), which is responsible for maintaining cell–cell adhesion and the stability of adhesive junctions. Furthermore, expression of the intermediate filament protein cytokeratin 18 (CK18) was consistently detected in both cell types (Figure 1c,e), confirming their epithelial-like nature.

### 3.2. Hypoxia-Induced Morphological Changes in Acomys and C57BL/6 Mesothelial Cells, Resulting in the Acquisition of Mesenchymal-like Characteristics

**Figure 2:**
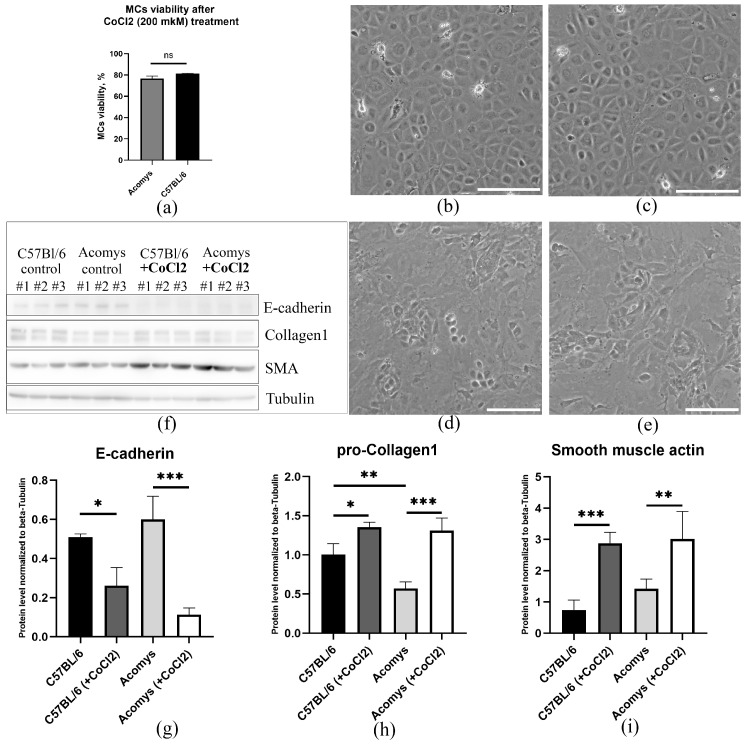
CoCl_2_-induced hypoxia induces fibroblast-like changes in MCs from the hearts of *Acomys cahirinus* and C57BL/6 mice. (**a**) Graph showing quantitative assessment of survival of epicardial MCs in *Acomys* and C57BL/6 mice after CoCl_2_ treatment (200 µM). (**b**,**c**) Representative phase-contrast images of MCs obtained from *Acomys cahirinus* (**b**) and C57BL/6 mice (**c**) cultivated under normoxia for 72 h. Scale bar = 100 μm. (**d**,**e**) Three-day hypoxia (treatment with 200 µM CoCl_2_) induces fibroblast-like morphology in MCs from the hearts of *Acomys cahirinus* (**d**) and C57BL/6 (**e**). Representative phase-contrast images show morphological changes in MCs. Scale bar = 100 μm. (**f**) Immunoblotting data showing the expression of E-cadherin, collagen I, α-SMA, and tubulin proteins in MCs obtained from the hearts of *Acomys cahirinus* and C57BL/6 mice under control conditions and after CoCl_2_ treatment. The labels “#1, #2, #3” refer to the numbers of the independent experimental replicates (original images can be found in Figure S3); (**g**) Graphs showing expression levels of E-cadherin, (**h**) pro-collagen I, and (**i**) α-SMA in MCs isolated from the hearts of *Acomys cahirinus* and C57BL/6 mice in the control group and following CoCl_2_ treatment (200 µM, 72 h). All data are expressed as mean ± standard deviation from at least three independent experiments. Statistical analysis was performed using one-way ANOVA with Tukey’s post hoc test. * *p* < 0.05, ** *p* < 0.01, *** *p* < 0.001.

Numerous studies have shown that hypoxia plays a key role in MCs activation and phenotypic modulation. The response of epicardial MCs to hypoxia was investigated using a chemically induced in vitro model based on CoCl_2_ treatment. This model relies on the capacity of Co^2+^ to substitute for Fe^2+^ in prolyl hydroxylases, which are crucial in mediating hypoxia-inducible factor (HIF) subunit degradation, resulting in HIF stabilization even under normoxic conditions [40]. Initial screening revealed a decrease in cell viability with increasing concentration of cobalt salts up to 500 µM (Supplementary Figure S1). Based on these data, 200 µM CoCl_2_ was selected for experiments assessing morphological changes and the expression of fibroblast-specific markers in MCs, as this concentration ensured moderate cell culture viability (Figure 2a).

Concurrently, our investigation revealed that hypoxia instigated morphological alterations in MCs from both *Acomys* and laboratory mice. Using phase-contrast microscopy, we found that hypoxia caused a change in the morphology of both cell types (Figure 2b–e). Cells cultured under normoxic conditions retained a polygonal or cobblestone shape (Figure 2b,c), whereas exposure to hypoxia induced spindle-shaped, fibroblast-like morphology (Figure 2d,e). Furthermore, hypoxia led to a decrease in the expression of epithelial cell marker E-cadherin (Figure 2f,g), accompanied by an increase in pro-collagen 1 and α-SMA expression (Figure 2f,h,i), as confirmed by immunoblotting. Thus, in vitro, MCs from *Acomys* and C57BL/6 exhibited a comparable response to CoCl_2_-induced hypoxia in vitro, characterized by changes in cell morphology, loss of epithelial characteristics, and acquisition of fibroblast-like features.

### 3.3. Myocardial Infarction Induced Comparable Levels of Hypoxia in Cardiac Tissue and Signs of a Profibrotic Response in Acomys and C57BL/6 Mice

**Figure 3:**
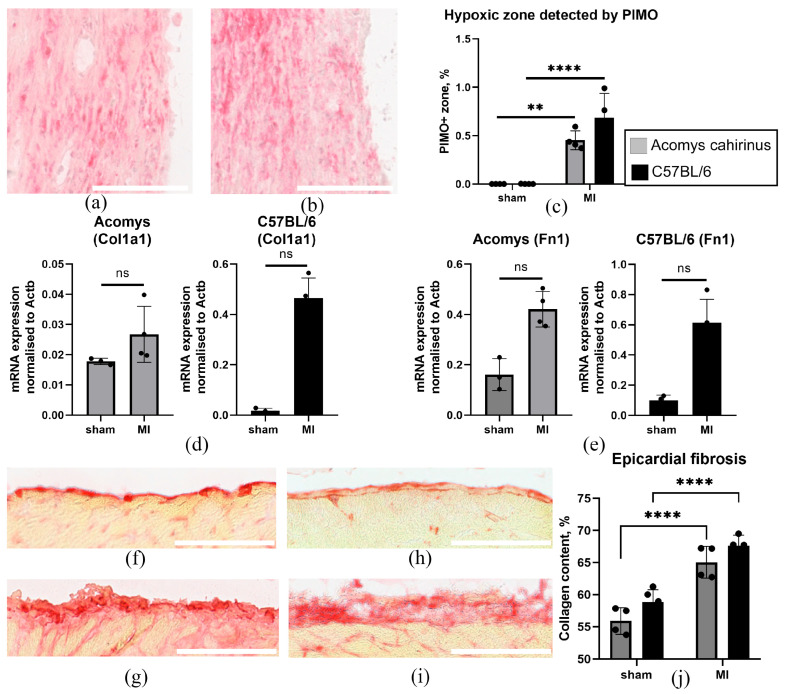
Myocardial infarction induces similar levels of hypoxia and profibrotic transformation of the epicardial mesothelium in *Acomys cahirinus* and C57BL/6 hearts by day 3 of repair. (**a**,**b**) Representative immunohistochemical staining of *Acomys cahirinus* (**a**) and C57BL/6 (**b**) heart cryosections with antibodies against pimonidazole adducts (red). Scale bar = 100 μm. (**c**) Graph quantifying the area of PIMO+ hypoxic zones in the hearts of *Acomys cahirinus* and C57BL/6 (3 days after MI). (**d**,**e**) Analysis of *Col1a1* and *Fn1* gene expression. Total RNA of epicardial MCs was isolated from the hearts of *Acomys cahirinus* and C57BL/6 on day 3 after MI, and specific mRNA levels were quantified by qRT-PCR, as described in Section 2.5. The data are normalized to *Actb*. Statistical analysis was performed using the Mann–Whitney U test (MCs from *Acomys cahirinus*: sham, n = 3; MI day 3, n = 4; MCs from C57BL/6: sham, n = 3; MI day 3, n = 4). (**f–i**) Representative images of C57BL/6 ((**f**) sham; (**g**) MI day 3) and *Acomys cahirinus* ((**h**) sham; (**i**) MI day 3) heart cryosections stained with Picrosirius red (red). Scale bar = 150 μm. (**j**) Graph quantifying collagen content in the epicardial regions of *Acomys cahirinus* and C57BL/6 (3 days after MI). All data are presented as mean ± standard deviation from at least three animals per group. Statistical analysis was performed using two-way ANOVA with Tukey’s post hoc test. ** *p* < 0.01, **** *p* < 0.0001.

Hypoxia following myocardial infarction is the primary cause of myocardial damage and impaired cardiac function. To assess the prevalence and localization of hypoxia, we visualized pimonidazole adducts. The accumulation of these adducts by cells has been shown to correlate strongly with low pO_2_ (<10 mmHg), as measured directly using microelectrodes [41,42] and by HIF-1α staining [43]. Consequently, this method provides information about the spatial localization of hypoxia in the heart. To compare the prevalence of hypoxia in *Acomys* and C57BL/6 heart tissue, we detected pimonidazole adducts 3 days after myocardial infarction (Figure 3a–c). Morphometric analysis demonstrated that ligation of the coronary artery in animals of both species resulted in the development of extensive hypoxic areas, affecting both the epicardium and the myocardium (Figure 3a,b). To assess the early post-infarction profibrotic response of the epicardial mesothelium, we isolated epicardial MCs from *Acomys* and C57BL/6 mice and analyzed the expression of *Col1a1* and *Fn1* genes using real-time PCR. We found that in MCs from both species, a trend toward increased expression of *Col1a1* and *Fn1* was observed on day 3 after MI, although these differences did not reach statistical significance compared to the sham-operated groups (Figure 3d,e). At the same time, Picrosirius staining revealed that collagen content in the epicardial zones of both *Acomys* and C57BL/6 was significantly higher than in sham-operated animals, with no significant difference between the two species (Figure 3f–j).

### 3.4. Myocardial Infarction Induced Comparable Dynamics of Epicardial Activation and Profibrotic Remodeling in Both Acomys and C57BL/6 Mice

**Figure 4:**
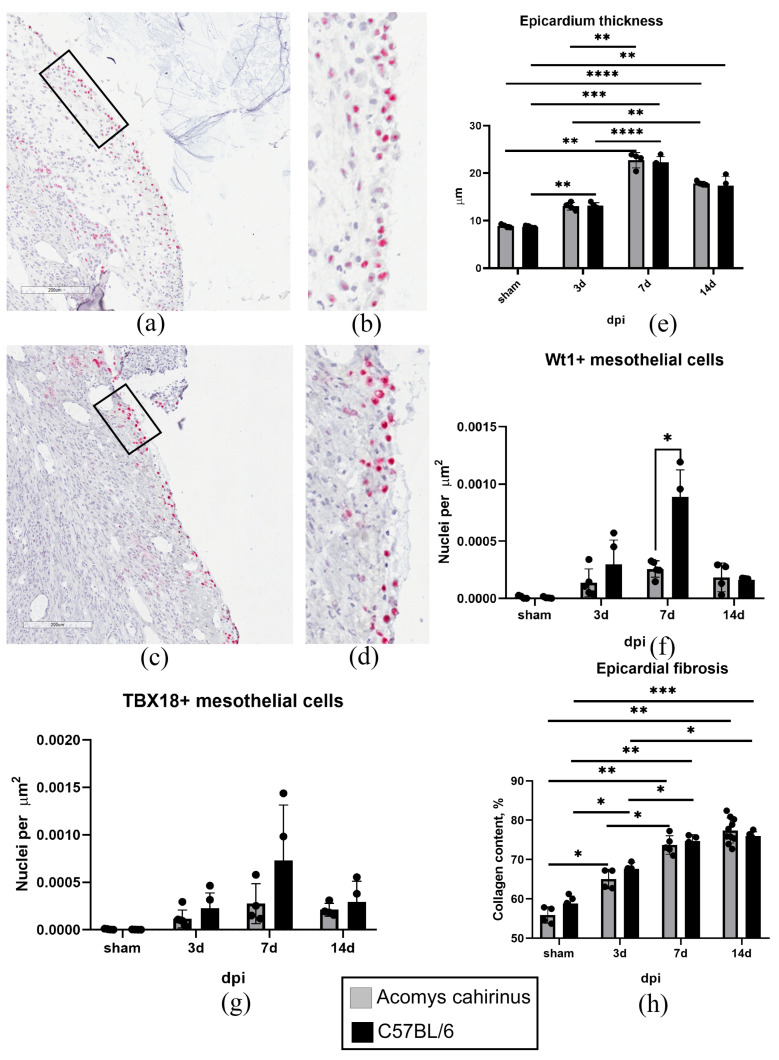
Hypoxia following MI induces comparable levels of MC activation and profibrotic remodeling of the epicardial regions in *Acomys cahirinus* and C57BL/6 hearts. (**a–d**) Representative immunohistochemical staining of *Acomys cahirinus* (**a**,**b**) and C57BL/6 (**c**,**d**) heart cryosections with antibodies against WT1 (red). (**b**,**d**) Enlarged views of the areas indicated by rectangles in (**a**) and (**c**), respectively. Scale bar = 100 μm. (**e–i**) Graphs quantifying epicardial thickness (**e**), amount of WT1+ (f), TBX18+ cells (**g**), and collagen content (**h**) in the epicardial regions of *Acomys cahirinus* and C57BL/6 hearts. All data are expressed as mean ± standard deviation from at least four animals per group. Statistical analysis was performed using two-way ANOVA with Tukey’s post hoc test. * *p* < 0.05, ** *p* < 0.01, *** *p* < 0.001, **** *p* < 0.0001.

Detection of pimonidazole adducts in the epicardial zone indicated local hypoxia (Figure 3a–c). This finding prompted us to further investigate how the MC activity changes in animals of both species. From day 3 after the infarction, we observed cell disorganization and an increase in the thickness of the epicardial layer, indicating MC activation (Figure 4a–e). Indeed, the mean thickness of the epicardium in animals of both species was significantly greater than in the intact myocardium (Figure 4e). Concurrently, epicardial thickness on days 3, 7 and 14 post-MI did not differ substantially between the two species (Figure 4e). Immunohistochemical studies revealed an accumulation of Wt1^+^ and TBX18^+^ epicardial progenitor cells (Figure 4a–d,f,g), indicating the entry of MCs into EMT and differentiation towards a fibroblast lineage [44,45,46]. In the early stages post-infarction, the number of detectable Wt1^+^ and TBX18^+^ cells significantly exceeded control values, increased during the first week, and then showed a downward trend (Figure 4f,g). It is noteworthy that a number of Wt1^+^ cells in C57BL/6 was significantly higher on day 7 post-MI in comparison with *Acomys cahirinus* (Figure 4f). However, collagen accumulation was similar in the epicardial region of both species during the 14-day period following the MI (Figure 4h). Thus, at different phases following myocardial infarction, *Acomys* and C57BL/6 mice exhibited comparable levels of epicardial MC activation, resulting in thickening of the epicardial layer, the formation of Wt1^+^ and TBX18^+^ fibroblast precursor cells, and collagen accumulation.

## 4. Discussion

Recent studies have significantly advanced our understanding of the role of epicardial MCs in maintaining tissue homeostasis and participating in the profibrotic reparative response to damage. Given the anatomical localization of this heterogeneous cell population, its interaction with various cardiac cell types, and production of biologically active components (cytokines, extracellular vesicles/exosomes, extracellular matrix proteins, adhesion molecules, etc.), several authors have conceptualized an “epicardial cell niche”. This specialized area performs a significant regulatory function and is subject to strict oversight [47]. Researchers have identified the oxygen content in the epicardial microenvironment as a critical factor in determining the fate of MCs [23,48]. It is now well established that partial oxygen pressure plays a dual role for MCs, functioning both as a metabolic substrate and as a regulator of critical functions associated with the EMT. This transition promotes differentiation, increased migration, proliferation, and secretion [49,50,51]. Conceptually, hypoxia causes the stabilization of HIF transcription factors, which bind to hypoxia response elements (HREs) to regulate genes involved in EMT, angiogenesis, and metabolic adaptation [24,52,53]. Epicardial activation is a complex process involving multiple signaling cascades, including SMAD, TGFβ, and Notch pathways [25,54,55] that drive the proliferation and differentiation of cells into fibroblasts and vascular smooth muscle cells. These pathways interact to coordinate the epicardium’s responses to injury and developmental signals, with hypoxia serving as a key upstream regulator. Understanding these mechanisms is imperative for elucidating the plasticity of the epicardium regarding the formation of the fibroblast/myofibroblast pool and its regenerative potential [56,57].

Of particular interest is studying the regulatory effects of physiological/pathological oxygen concentrations on MCs in animals with evolutionarily conserved hypoxia protection mechanisms and a high capacity for scar-free tissue repair. These include the African spiny mouse (*Acomys cahirinus*), a rodent from the family Muridae [58]. A remarkable property of this species is its ability to tolerate ischemia, which promotes cardiac cell survival, alters scar tissue structure, and increases vascularization of the damaged area. This approach has been shown to result in a significant reduction in pathological remodeling and improved survival rates in animals following experimental myocardial infarction [31,32,33]. In this study, we used in vitro and in vivo models to assess the response of *Acomys* epicardial MCs to hypoxia, a known consequence of myocardial infarction. We isolated *Acomys* MCs and demonstrated in vitro that they retain epithelial-like characteristics (Figure 1a,b) and express specialized mesothelial markers under normoxic conditions (Figure 1c–e). These include E-cadherin, a specialized protein that plays a key role in the adhesion of epithelial-like cells. This calcium-sensitive homotypic adhesion is the primary stabilizing interaction in the intercellular adhesion, as well as a signal for cell polarization and differentiation [37,59,60,61]. The second characteristic protein we identified in the mesothelium was cytokeratin 18 [62,63], an intermediate filament protein of the keratin family. This protein plays a crucial role in maintaining the structural integrity of the epithelial barrier and is a well-established marker for tracking the fate of MCs of different origins [64,65].

Cobalt chloride-induced hypoxic stress did not increase proliferation in MCs from both *Acomys* and conventional laboratory mice (Figure 2a, Supplementary Figure S1). Concurrently, MCs from both species showed analogous responses to hypoxic exposure, manifesting signs of morphological alterations (Figure 2b–e) and increased production of fibroblast-specific proteins (Figure 2f,h,i). It is widely acknowledged that decreased oxygen levels function as a key integrative factor in preserving homeostasis across diverse niches [66,67], modulating cell self-renewal and generating committed progeny. MCs are no exception; a decrease in oxygen concentration significantly alters their properties, likely through the activation of HIF signaling. Numerous studies demonstrate marked activation of HIF—heterodimeric transcription factors consisting of α and constitutive β subunits—in MCs of various species, including mice, zebrafish, rats, and humans, highlighting their important role as central mediators of the hypoxic response [24,25,68]. The variability in the expression of the oxygen-sensitive α subunits is determined by the functionality of the HIF-1α and HIF-2α protein isoforms [69]. Under normoxia, prolyl hydroxylases catalyze the hydroxylation of specific proline residues on the α subunit, marking it for degradation via the ubiquitin–proteasome pathway. Under reduced oxygen levels, prolyl hydroxylase activity is inhibited, allowing the α subunit to translocate to the nucleus and bind to HREs, regulating the transcription of a variety of target genes [70]. Furthermore, the activation/stabilization of HIFs through coordinated interaction with the Notch, TGFβ, and BMP signaling systems regulates epicardial EMT, differentiation, and progenitor cell activation [25,54,55,56,71,72]. It has been established that exposure to CoCl_2_-induced hypoxia results in a partial loss of E-cadherin expression (Figure 2f,g), leading to impaired intercellular adhesion of MCs and activation of matrix metalloproteinases [73]. This phenomenon may result from HIF-dependent regulation of transcription factors [74,75,76,77] (TWIST, SNAIL, SLUG, SIP1, and ZEB1) and the activation of alternative signaling mechanisms [75,78,79]. This process ultimately leads to cell division associated with the reorganization of the actin cytoskeleton, resulting in the formation of fibroblast-like cells that express mesenchymal markers such as pro-collagen 1, α-SMA, and vimentin de novo [80]. Furthermore, following hypoxic exposure, the epicardial mesothelium of both species exhibited a mesenchymal-like morphology and augmented pro-collagen expression, as well as increased α-SMA expression. This phenomenon may be attributed to the direct transcriptional upregulation of *Col1a1* (collagen I) and *Acta2* (α-SMA) genes facilitated by HIF [78].

Another important finding of this study concerns the in vivo characterization of the response of *Acomys* epicardial mesothelium to the acute ischemic injury (Figure 3 and Figure 4). It is well established that the epicardial MCs can be reactivated following injury, leading to the initiation of an embryonic program and the acquisition of pro-regenerative properties [81,82]. Upon activation, a subset of MCs proliferates and undergoes EMT, giving rise to epicardial progenitor cells. The resulting Wt1^+^, Tbx18^+^, and Tcf21^+^ progenitor cells migrate into the underlying myocardium and contribute to several different cardiac cell lineages, including fibroblasts [83,84,85,86,87]. Although no statistically significant differences were observed compared with the sham-operated groups, there was a tendency toward increased *Col1a1* and *Fn1* expression in MCs from both *Acomys* and C57BL/6 mice on day 3 post-MI (Figure 3d,e). Picrosirius staining revealed significantly higher collagen content in the epicardial zones of both *Acomys* and C57BL/6 mice on day 3 post-MI compared with sham-operated animals (Figure 3f–j). Notably, there was no significant difference in epicardial collagen deposition between these two species, indicating a similar profibrotic response in the MC derivatives. Furthermore, these cells secrete a wide range of paracrine factors [88,89], which modulate the healing and repair of the heart muscle. Their expression is mediated by hypoxia, which includes the activation of Wt1 and TBX18 promoters due to the presence of HREs binding sites for HIF-1α [26,90,91,92]. It is important to note that Wt1 and Tbx18 are expressed in the early stages of profibrotic differentiation of epicardial MCs. These markers are primarily expressed in epicardial and interstitial fibrosis, but not in perivascular fibrosis, making them reliable indicators of fibrogenic remodeling of heart tissue [43,93]. Using an in vivo myocardial infarction model, we found similar rates of hypoxia prevalence (detected based on pimonidazole adducts) in *Acomys cahirinus* and *Mus musculus* (Figure 3a–c) and comparable dynamics of increased activity of Wt1^+^ and Tbx18^+^ epicardial MCs (Figure 4f,g). The higher number of Wt1^+^ cells in C57BL/6 mice on day 7 post-MI, compared to *Acomys* mice, is likely due to the species-specific differences in activation pathways and the multifunctionality of the epicardial derivative. This could be associated with changes in the hypoxia levels, the formation of a gradient of factors (e.g., transforming growth factor beta, brain natriuretic peptide [94,95], etc.), both of which determine damaged area revascularization, differences in the composition and stiffness of the extracellular matrix, interactions with distinct patterns of inflammatory cells, and other factors.

Acting individually or in combination, these stimuli can regulate the fate of Wt1^+^ epicardial mesothelial cells, promoting the formation of a fibroblast/myofibroblast pool and specific c-kit^+^ vasculogenic resident precursors [96,97]. Furthermore, these cells have been shown to subsequently differentiate into cardiomyocytes and coronary endothelial cells in the infarct zone [87,98]. It can be assumed that, as fibrosis progresses, Wt1^+^ cells respond to activating stimuli by ECM turnover via matrix metalloproteinases and their inhibitors, with their contribution being determined by their specific cell type [99]. It is important to note that the early post-infarction remodeling of the *Acomys* epicardium has not been previously studied. The Bartscherer group [32] investigated the potential for complete recovery of the epicardium in *Acomys* spp. following hypoxic exposure 100 days post- MI. Despite an increased number of DDAH2^+^ (dimethylarginine dimethylaminohydrolase 2) cells in the epicardial zone of *Acomys* compared to control laboratory mice, the authors examined only the late phase of post-infarction recovery and did not compare epicardial activation directly with indicators of repair or early improvement in cardiac function.

We demonstrated that acute hypoxia activated *Acomys cahirinus* epicardial MCs, leading to the formation of a pool of Wt1^+^ and TBX18^+^ epicardial progenitor cells. This phenomenon has been observed in a variety of species, including mice [23], salamanders [28,100], and zebrafish [27,101]. Furthermore, the formation of progenitor cells in both species was accompanied by collagen accumulation in the activated epicardial zone. This finding suggests that the profibrotic response of the epicardial mesothelium to hypoxia is highly conserved. It is important to note that epicardial derivatives with increased collagen expression may differ from those that formed massively in the interstitium after ischemic injury. It is possible that they promote repair via a distinct mechanism, similar to zebrafish [102].

The present study has several limitations that offer opportunities for further research. First, we used animals of different chronological ages: C57BL/6 mice at 16–18 weeks and *Acomys cahirinus* at 18–20 weeks. While these age ranges were selected to approximate young adult equivalents based on known lifespans and sexual maturity of each species, the lack of age matching in absolute terms may introduce confounding variables. Differences in metabolic rate, hormonal status, or tissue maturation between 16 and 18 weeks in C57BL/6 and 18–20 weeks in *Acomys* could theoretically affect the extent of epicardial activation, fibrotic remodeling, or hypoxia tolerance following myocardial infarction. Future studies employing cross-species age calibration would help clarify whether the observed responses are truly species-specific or partially age-dependent.

The methodological limitations include the limited availability of specialized antibodies against *Acomys* mesothelial surface markers. This restricts the isolation and characterization of individual *Acomys* MC subpopulations. A further issue is the inability to perform lineage tracing. This has prevented us from tracking the final fate of epicardial-derived cells and their contribution to *Acomys* myocardial regeneration. Furthermore, the absence of spatial transcriptomic assessment excludes the correlation of changes in the cellular transcriptome with the hypoxia gradient in tissues. A key issue that requires further study is the analysis of the secretory profile of *Acomys* MCs, which may be determined by a unique repertoire of paracrine factors. This likely encompasses not only classical growth factors but also a range of extracellular vesicles carrying conserved pro-regenerative microRNAs, as well as hypoxia-sensitive proteins and extracellular matrix components. Further experimental testing of this hypothesis will validate our data and may allow us to modulate epicardial niche activity to develop new therapeutic strategies for stimulating cardiac repair.

## 5. Conclusions

In this study, we have isolated and characterized the epicardial MCs of the African spiny mouse (*Acomys cahirinus*). This species is a unique model animal with a high cardiac repair capacity. We demonstrated that their response to hypoxic stress in vitro (i.e., lack of proliferation, morphological changes, and increased production of type I collagen and α-SMA) is not fundamentally different from the response of cells derived from laboratory C57Bl/6 mice. In vivo, *Acomys* and *Mus musculus* exhibited comparable epicardial activation, including similar dynamics of epicardial activation and collagen accumulation in the surface layer of the heart. This finding suggests that the initiation of a profibrotic repair program in the epicardial mesothelium in response to damage is an evolutionarily conserved mechanism. We propose that a key direction for further research should be the analysis of the specific populations of *Acomys* epicardial mesothelial-derived cells and their secretory profile, which will help to explain the pro-regenerative properties of this species. The elucidation of these mechanisms has the potential to offer new avenues for the therapeutic modulation of the epicardial niche.

## Figures and Tables

**Table 1:** Primers.

Gene Name	Forward	Reverse
acCol1a1	TGGACCCAAGGGTACTGCT	GAACACCACGCTCTCCAGAC
acFn1	CACCAACGAACTTGCACCTG	GCAGGAACTCTGGTCAGCAT
acActb	TCGTTCACCGCAAATGCTTC	GCCTTCACCGTTCCAGTTTTT
mCol1a1	CCGCTGGTCAAGATGGTC	CTCCAGCCTTTCCAGGTTCT
mFn1	GGAATGGACCTGCAAACCTA	GTAGGGCTTTTCCCAGGTCT
mActb	GGCTGTATTCCCCTCCATCG	CCAGTTGGTAACAATGCCATGT

## Data Availability

All data related to this work can be made available upon request to the corresponding authors.

## Supplementary Materials

The following supporting information can be downloaded at: https://www.mdpi.com/article/10.3390/biom16050717/s1, Figure S1: Graph showing quantitative assessment of survival of cardiac MCs from Acomys and C57BL/6 mice after CoCl_2_ treatment; Figure S2: Original immunoblotting images showing the expression of E-cadherin, cytokeratin, and tubulin proteins in MCs obtained from the hearts of Acomys cahirinus and C57BL/6 mice; Figure S3: Original immunoblotting images showing the expression of E-cadherin, collagen I, α-SMA, and tubulin proteins in MCs obtained from the hearts of Acomys cahirinus and C57BL/6 mice under control conditions and after CoCl_2_ treatment.

## Abbreviations

The following abbreviations are used in this manuscript:

MI	Myocardial infarction
EMT	Epithelial–mesenchymal transition
HIF	Hypoxia-inducible factors
ECM	Extracellular matrix
HRE	Hypoxia response elements
PBS	Phosphate-buffered saline
MCs	Mesothelial cells

## References

[1] Irlik K., Piaśnik J., Hendel M., Faron U., Lip G.Y.H., Nabrdalik K., Prokopidis K. (2026). Mortality and Heart Failure Hospitalizations in Heart Failure with Preserved Ejection Fraction Compared to Heart Failure with Reduced Ejection Fraction: A Systematic Review and Meta-Analysis. ESC Heart Fail..

[2] Nabel E.G., Braunwald E. (2012). A Tale of Coronary Artery Disease and Myocardial Infarction. N. Engl. J. Med..

[3] Bergmann O., Zdunek S., Felker A., Salehpour M., Alkass K., Bernard S., Sjostrom S.L., Szewczykowska M., Jackowska T., dos Remedios C. (2015). Dynamics of Cell Generation and Turnover in the Human Heart. Cell.

[4] Secco I., Giacca M. (2023). Regulation of Endogenous Cardiomyocyte Proliferation: The Known Unknowns. J. Mol. Cell. Cardiol..

[5] Rochette L., Malka G., Cottin Y. (2017). Hypoxia and Heart Regeneration: A New Paradoxical Approach for Cardioprotection. Arch. Cardiovasc. Dis..

[6] Wolff L., Wolff R. (1965). Diseases of the Pericardium. Annu. Rev. Med..

[7] Sanchez-Fernandez C., Rodriguez-Outeiriño L., Matias-Valiente L., Ramirez De Acuña F., Hernandez-Torres F., Lozano-Velasco E., Dominguez J.N., Franco D., Aranega A.E. (2022). Regulation of Epicardial Cell Fate during Cardiac Development and Disease: An Overview. Int. J. Mol. Sci..

[8] Moore-Morris T., Guimarães-Camboa N., Yutzey K.E., Pucéat M., Evans S.M. (2015). Cardiac Fibroblasts: From Development to Heart Failure. J. Mol. Med..

[9] Bakalenko N., Kuznetsova E., Dergilev K., Beloglazova I., Malashicheva A. (2026). Mesothelial Cells in Fibrosis: Focus on Intercellular Crosstalk. Biomolecules.

[10] Gittenberger-de Groot A.C., Vrancken Peeters M.-P.F.M., Mentink M.M.T., Gourdie R.G., Poelmann R.E. (1998). Epicardium-Derived Cells Contribute a Novel Population to the Myocardial Wall and the Atrioventricular Cushions. Circ. Res..

[11] Gittenberger-de Groot A.C., Vrancken Peeters M.-P.F.M., Bergwerff M., Mentink M.M.T., Poelmann R.E. (2000). Epicardial Outgrowth Inhibition Leads to Compensatory Mesothelial Outflow Tract Collar and Abnormal Cardiac Septation and Coronary Formation. Circ. Res..

[12] Zamora M., Männer J., Ruiz-Lozano P. (2007). Epicardium-Derived Progenitor Cells Require β-Catenin for Coronary Artery Formation. Proc. Natl. Acad. Sci. USA.

[13] Pérez-Pomares J.M., De La Pompa J.L. (2011). Signaling During Epicardium and Coronary Vessel Development. Circ. Res..

[14] Silva E.D., Pereira-Sousa D., Ribeiro-Costa F., Cerqueira R., Enguita F.J., Gomes R.N., Dias-Ferreira J., Pereira C., Castanheira A., Pinto-do-Ó P. (2024). Pericardial Fluid Accumulates microRNAs That Regulate Heart Fibrosis after Myocardial Infarction. Int. J. Mol. Sci..

[15] Oliveira C.C., Córdoba J., Pearson J.R., Guruceaga E., Marín-Sedeño E., López-Moreno M., Guadix J.A., García-Caballero M., Pérez-Pomares J.M., Ruiz-Villalba A. (2025). Revealing the Complexity of the Epicardial Secretome. Sci. Rep..

[16] Lavine K.J., Yu K., White A.C., Zhang X., Smith C., Partanen J., Ornitz D.M. (2005). Endocardial and Epicardial Derived FGF Signals Regulate Myocardial Proliferation and Differentiation in Vivo. Dev. Cell.

[17] Chen T.H.-P., Chang T.-C., Kang J.-O., Choudhary B., Makita T., Tran C.M., Burch J.B.E., Eid H., Sucov H.M. (2002). Epicardial Induction of Fetal Cardiomyocyte Proliferation via a Retinoic Acid-Inducible Trophic Factor. Dev. Biol..

[18] Duan J., Gherghe C., Liu D., Hamlett E., Srikantha L., Rodgers L., Regan J.N., Rojas M., Willis M., Leask A. (2012). Wnt1/Βcatenin Injury Response Activates the Epicardium and Cardiac Fibroblasts to Promote Cardiac Repair. EMBO J..

[19] Braitsch C.M., Combs M.D., Quaggin S.E., Yutzey K.E. (2012). Pod1/Tcf21 Is Regulated by Retinoic Acid Signaling and Inhibits Differentiation of Epicardium-Derived Cells into Smooth Muscle in the Developing Heart. Dev. Biol..

[20] Bollini S., Vieira J.M.N., Howard S., Dubè K.N., Balmer G.M., Smart N., Riley P.R. (2014). Re-Activated Adult Epicardial Progenitor Cells Are a Heterogeneous Population Molecularly Distinct from Their Embryonic Counterparts. Stem Cells Dev..

[21] Ruiz-Villalba A., Guadix J.A., Pérez-Pomares J.M. (2024). Epicardium and Coronary Vessels. Adv. Exp. Med. Biol..

[22] Dergilev K.V., Komova A.V., Tsokolaeva Z.I., Beloglazova I.B., Parfyonova Y.V. (2020). Epicardium as a New Target for Regenerative Technologies in Cardiology. Genes Cells.

[23] Hesse J., Owenier C., Lautwein T., Zalfen R., Weber J.F., Ding Z., Alter C., Lang A., Grandoch M., Gerdes N. (2021). Single-Cell Transcriptomics Defines Heterogeneity of Epicardial Cells and Fibroblasts within the Infarcted Murine Heart. eLife.

[24] Gamen E., Price E.L., Pezzolla D., De Villiers C., Gunadasa-Rohling M., Lokman A.B., Cosma M.-A., Sayers J., Silva C.R., Salama R. (2025). Stabilisation of HIF Signalling in the Mouse Epicardium Extends Embryonic Potential and Neonatal Heart Regeneration. eLife.

[25] Tao J., Barnett J.V., Watanabe M., Ramírez-Bergeron D. (2018). Hypoxia Supports Epicardial Cell Differentiation in Vascular Smooth Muscle Cells through the Activation of the TGFβ Pathway. J. Cardiovasc. Dev. Dis..

[26] Jing X., Gao Y., Xiao S., Qin Q., Wei X., Yan Y., Wu L., Deng S., Du J., Liu Y. (2016). Hypoxia Induced the Differentiation of Tbx18-Positive Epicardial Cells to CoSMCs. Sci. Rep..

[27] González-Rosa J.M., Peralta M., Mercader N. (2012). Pan-Epicardial Lineage Tracing Reveals That Epicardium Derived Cells Give Rise to Myofibroblasts and Perivascular Cells during Zebrafish Heart Regeneration. Dev. Biol..

[28] Eroglu E., Yen C.Y.T., Tsoi Y.-L., Witman N., Elewa A., Joven Araus A., Wang H., Szattler T., Umeano C.H., Sohlmér J. (2022). Epicardium-Derived Cells Organize through Tight Junctions to Replenish Cardiac Muscle in Salamanders. Nat. Cell Biol..

[29] Sandoval A.G.W., Maden M. (2020). Regeneration in the Spiny Mouse, Acomys, a New Mammalian Model. Curr. Opin. Genet. Dev..

[30] Allen R.S., Seifert A.W. (2025). Spiny Mice (*Acomys*) Have Evolved Cellular Features to Support Regenerative Healing. Ann. N. Y. Acad. Sci..

[31] Peng H., Shindo K., Donahue R.R., Gao E., Ahern B.M., Levitan B.M., Tripathi H., Powell D., Noor A., Elmore G.A. (2021). Adult Spiny Mice (*Acomys*) Exhibit Endogenous Cardiac Recovery in Response to Myocardial Infarction. npj Regen. Med..

[32] Koopmans T., Van Beijnum H., Roovers E.F., Tomasso A., Malhotra D., Boeter J., Psathaki O.E., Versteeg D., Van Rooij E., Bartscherer K. (2021). Ischemic Tolerance and Cardiac Repair in the Spiny Mouse (Acomys). npj Regen. Med..

[33] Qi Y., Dasa O., Maden M., Vohra R., Batra A., Walter G., Yarrow J.F., Aranda J.M., Raizada M.K., Pepine C.J. (2021). Functional Heart Recovery in an Adult Mammal, the Spiny Mouse. Int. J. Cardiol..

[34] Dergilev K., Tsokolaeva Z., Goltseva Y., Beloglazova I., Ratner E., Parfyonova Y. (2023). Urokinase-Type Plasminogen Activator Receptor Regulates Prosurvival and Angiogenic Properties of Cardiac Mesenchymal Stromal Cells. Int. J. Mol. Sci..

[35] Dergilev K., Beloglazova I., Tsokolaeva Z., Azimova E., Dolgodvorova A., Goltseva Y., Boldyreva M., Menshikov M., Penkov D., Parfyonova Y. (2025). HGF Overexpression in Mesenchymal Stromal Cell-Based Cell Sheets Enhances Autophagy-Dependent Cytoprotection and Proliferation to Guard the Epicardial Mesothelium. Int. J. Mol. Sci..

[36] Goltseva Y., Tsokolaeva Z., Iarushkina I., Beloglazova I., Boldyreva M., Ratner E., Parfyonova Y., Dergilev K. (2025). The 3D World of Spheroids: Searching for an Optimal Method of Fabricating Pro-Reparative Cardiospheres. Int. J. Mol. Sci..

[37] Dergilev K.V., Goltseva Y.D., Tsokolaeva Z.I., Beloglazova I.B., Yarushkina I.S., Azimova E.D., Ratner E.I., Parfenova E.V. (2025). Autophagy activity in cardiac fibroblasts at the early stages of cardiac dysfunction induced by pressure overload. Russ. Cardiol. Bull..

[38] Dergilev K., Zubko A., Beloglazova I., Tsokolaeva Z., Azimova E., Dolgodvorova A., Iarushkina I., Andreev A., Shiryaev A., Docshin P. (2025). Human Pericardial Fluid-Derived Cells Exhibit Mesothelial-like Properties and Exert Proangiogenic Effects on Endothelial Cells. Cells.

[39] Quijada P., Trembley M.A., Misra A., Myers J.A., Baker C.D., Pérez-Hernández M., Myers J.R., Dirkx R.A., Cohen E.D., Delmar M. (2021). Coordination of Endothelial Cell Positioning and Fate Specification by the Epicardium. Nat. Commun..

[40] Muñoz-Sánchez J., Chánez-Cárdenas M.E. (2019). The Use of Cobalt Chloride as a Chemical Hypoxia Model. J. Appl. Toxicol..

[41] Gross M.W., Karbach U., Groebe K., Franko A.J., Mueller-Klieser W. (1995). Calibration of Misonidazole Labeling by Simultaneous Measurement of Oxygen Tension and Labeling Density in Multicellular Spheroids. Int. J. Cancer.

[42] Ow C.P.C., Ullah M.M., Ngo J.P., Sayakkarage A., Evans R.G. (2019). Detection of Cellular Hypoxia by Pimonidazole Adduct Immunohistochemistry in Kidney Disease: Methodological Pitfalls and Their Solution. Am. J. Physiol.-Ren. Physiol..

[43] Swartz J.E., Smits H.J.G., Philippens M.E.P., De Bree R., Kaanders J.H.A.M., Willems S.M. (2022). Correlation and Colocalization of HIF-1α and Pimonidazole Staining for Hypoxia in Laryngeal Squamous Cell Carcinomas: A Digital, Single-Cell-Based Analysis. Oral Oncol..

[44] Braitsch C.M., Kanisicak O., Van Berlo J.H., Molkentin J.D., Yutzey K.E. (2013). Differential Expression of Embryonic Epicardial Progenitor Markers and Localization of Cardiac Fibrosis in Adult Ischemic Injury and Hypertensive Heart Disease. J. Mol. Cell. Cardiol..

[45] Braitsch C., Yutzey K. (2013). Transcriptional Control of Cell Lineage Development in Epicardium-Derived Cells. J. Dev. Biol..

[46] Ruiz-Villalba A., Simón A.M., Pogontke C., Castillo M.I., Abizanda G., Pelacho B., Sánchez-Domínguez R., Segovia J.C., Prósper F., Pérez-Pomares J.M. (2015). Interacting Resident Epicardium-Derived Fibroblasts and Recruited Bone Marrow Cells Form Myocardial Infarction Scar. J. Am. Coll. Cardiol..

[47] Sanchez-Fernandez C., Rodriguez-Outeiriño L., Matias-Valiente L., Ramírez De Acuña F., Franco D., Aránega A.E. (2023). Understanding Epicardial Cell Heterogeneity during Cardiogenesis and Heart Regeneration. J. Cardiovasc. Dev. Dis..

[48] Sayed A., Turoczi S., Soares-da-Silva F., Marazzi G., Hulot J.-S., Sassoon D., Valente M. (2022). Hypoxia Promotes a Perinatal-like Progenitor State in the Adult Murine Epicardium. Sci. Rep..

[49] Morishita Y., Ookawara S., Hirahara I., Muto S., Nagata D. (2016). HIF-1α Mediates Hypoxia-Induced Epithelial–Mesenchymal Transition in Peritoneal Mesothelial Cells. Ren. Fail..

[50] Dergilev K.V., Tsokolaeva Z.I., Beloglazova I.B., Ratner E.I., Parfenova E.V. (2021). Transforming Growth Factor Beta (TGF-Β1) Induces Pro-Reparative Phenotypic Changes in Epicardial Cells in Mice. Bull. Exp. Biol. Med..

[51] Yang X., Bao M., Fang Y., Yu X., Ji J., Ding X. (2021). STAT3/HIF-1α Signaling Activation Mediates Peritoneal Fibrosis Induced by High Glucose. J. Transl. Med..

[52] Kocabas F., Mahmoud A.I., Sosic D., Porrello E.R., Chen R., Garcia J.A., DeBerardinis R.J., Sadek H.A. (2012). The Hypoxic Epicardial and Subepicardial Microenvironment. J. Cardiovasc. Transl. Res..

[53] Zhang Y., Qin X., Guo R., Sun X., Zhao Z., Guo H., Wang M., Li S., Li T., Lv D. (2025). Notch-1 Regulates Collective Breast Cancer Cell Migration by Controlling Intercellular Junction and Cytoskeletal Organization. Cell Prolif..

[54] Ghosh A.K., Crake T., Manisty C., Westwood M. (2018). Pericardial Disease in Cancer Patients. Curr. Treat. Options Cardiovasc. Med..

[55] Docshin P., Panshin D., Malashicheva A. (2024). Molecular Interplay in Cardiac Fibrosis: Exploring the Functions of RUNX2, BMP2, and Notch. Rev. Cardiovasc. Med..

[56] Li G., Yan Z., Han L., Wu S., Wang M., Qi A., Zhou Z., Wang N., Sun R., Zhou X. (2024). Research Progress on Epicardial Repair After Myocardial Injury. Cardiol. Rev..

[57] DeLaughter D.M., Clark C.R., Christodoulou D.C., Seidman C.E., Baldwin H.S., Seidman J.G., Barnett J.V. (2016). Transcriptional Profiling of Cultured, Embryonic Epicardial Cells Identifies Novel Genes and Signaling Pathways Regulated by TGFβR3 In Vitro. PLoS ONE.

[58] Okamura D.M., Nguyen E.D., Beier D.R., Majesky M.W. (2022). Wound Healing and Regeneration in Spiny Mice (Acomys Cahirinus). Current Topics in Developmental Biology.

[59] Takeichi M. (1995). Morphogenetic Roles of Classic Cadherins. Curr. Opin. Cell Biol..

[60] Takeichi M. (1991). Cadherin Cell Adhesion Receptors as a Morphogenetic Regulator. Science.

[61] Wada A.M., Smith T.K., Osler M.E., Reese D.E., Bader D.M. (2003). Epicardial/Mesothelial Cell Line Retains Vasculogenic Potential of Embryonic Epicardium. Circ. Res..

[62] Xu Y., Zhang X., Fu Z., Dong Y., Yu Y., Liu Y., Liu Z., Chen J., Yao Y., Chen Y. (2024). Intrapericardial Administration of Human Pericardial Fluid Cells Improves Cardiac Functions in Rats with Heart Failure. Stem Cells Dev..

[63] Peeters M.-P.F.M.V., Mentink M.M.T., Poelmann R.E., Groot A.C.G. (1995). Cytokeratins as a Marker for Epicardial Formation in the Quail Embryo. Anat. Embryol..

[64] Means A.L., Xu Y., Zhao A., Ray K.C., Gu G. (2008). A CK19^CreERT^ Knockin Mouse Line Allows for Conditional DNA Recombination in Epithelial Cells in Multiple Endodermal Organs. Genesis.

[65] Westcott G.P., Emont M.P., Li J., Jacobs C., Tsai L., Rosen E.D. (2021). Mesothelial Cells Are Not a Source of Adipocytes in Mice. Cell Rep..

[66] Reddan B., Cummins E.P. (2025). The Regulation of Cell Metabolism by Hypoxia and Hypercapnia. J. Biol. Chem..

[67] Morikawa T., Takubo K. (2016). Hypoxia Regulates the Hematopoietic Stem Cell Niche. Pflüg. Arch.-Eur. J. Physiol..

[68] Dunwoodie S.L. (2009). The Role of Hypoxia in Development of the Mammalian Embryo. Dev. Cell.

[69] Shawki H.H., Ammar A.Y., Mansour M., Minisy F.M. (2026). Divergent Roles of HIF-1α and HIF-2α in Embryonic Development and Early Pregnancy. Int. J. Mol. Sci..

[70] Parsad R., Bagiyal M., Malhotra S., Arora R., Ahlawat S. (2026). Molecular Architecture and Regulatory Dynamics of Hypoxia-Inducible Factors in Livestock: A Narrative Review. Int. J. Biol. Macromol..

[71] Nusrat O., Belotte J., Fletcher N.M., Memaj I., Saed M.G., Diamond M.P., Saed G.M. (2016). The Role of Angiogenesis in the Persistence of Chemoresistance in Epithelial Ovarian Cancer. Reprod. Sci..

[72] Docshin P.M., Karpov A.A., Mametov M.V., Ivkin D.Y., Kostareva A.A., Malashicheva A.B. (2022). Mechanisms of Regenerative Potential Activation in Cardiac Mesenchymal Cells. Biomedicines.

[73] Larue L., Antos C., Butz S., Huber O., Delmas V., Dominis M., Kemler R. (1996). A Role for Cadherins in Tissue Formation. Development.

[74] Higgins D.F., Kimura K., Iwano M., Haase V.H. (2008). Hypoxia-Inducible Factor Signaling in the Development of Tissue Fibrosis. Cell Cycle.

[75] Chen N., Chen X., Huang R., Zeng H., Gong J., Meng W., Lu Y., Zhao F., Wang L., Zhou Q. (2009). BCL-xL Is a Target Gene Regulated by Hypoxia-Inducible Factor-1α. J. Biol. Chem..

[76] Evans A.J., Russell R.C., Roche O., Burry T.N., Fish J.E., Chow V.W.K., Kim W.Y., Saravanan A., Maynard M.A., Gervais M.L. (2007). VHL Promotes E2 Box-Dependent E-Cadherin Transcription by HIF-Mediated Regulation of SIP1 and Snail. Mol. Cell. Biol..

[77] Huang C.-H., Yang W.-H., Chang S.-Y., Tai S.-K., Tzeng C.-H., Kao J.-Y., Wu K.-J., Yang M.-H. (2009). Regulation of Membrane-Type 4 Matrix Metalloproteinase by SLUG Contributes to Hypoxia-Mediated Metastasis. Neoplasia.

[78] Orphanides C., Fine L.G., Norman J.T. (1997). Hypoxia Stimulates Proximal Tubular Cell Matrix Production via a TGF-Β1-Independent Mechanism. Kidney Int..

[79] Schäffer L., Scheid A., Spielmann P., Breymann C., Zimmermann R., Meuli M., Gassmann M., Marti H.H., Wenger R.H. (2003). Oxygen-Regulated Expression of TGF-Β3, a Growth Factor Involved in Trophoblast Differentiation. Placenta.

[80] Strutz F., Okada H., Lo C.W., Danoff T., Carone R.L., Tomaszewski J.E., Neilson E.G. (1995). Identification and Characterization of a Fibroblast Marker: FSP1. J. Cell Biol..

[81] Peralta M., Steed E., Harlepp S., González-Rosa J.M., Monduc F., Ariza-Cosano A., Cortés A., Rayón T., Gómez-Skarmeta J.-L., Zapata A. (2013). Heartbeat-Driven Pericardiac Fluid Forces Contribute to Epicardium Morphogenesis. Curr. Biol. CB.

[82] Simões F.C., Riley P.R. (2018). The Ontogeny, Activation and Function of the Epicardium during Heart Development and Regeneration. Development.

[83] Streef T.J., Smits A.M. (2021). Epicardial Contribution to the Developing and Injured Heart: Exploring the Cellular Composition of the Epicardium. Front. Cardiovasc. Med..

[84] Martínez-Estrada O.M., Lettice L.A., Essafi A., Guadix J.A., Slight J., Velecela V., Hall E., Reichmann J., Devenney P.S., Hohenstein P. (2010). Wt1 Is Required for Cardiovascular Progenitor Cell Formation through Transcriptional Control of Snail and E-Cadherin. Nat. Genet..

[85] Kikuchi K., Gupta V., Wang J., Holdway J.E., Wills A.A., Fang Y., Poss K.D. (2011). *Tcf21+* Epicardial Cells Adopt Non-Myocardial Fates during Zebrafish Heart Development and Regeneration. Development.

[86] Smart N., Bollini S., Dubé K.N., Vieira J.M., Zhou B., Davidson S., Yellon D., Riegler J., Price A.N., Lythgoe M.F. (2011). De Novo Cardiomyocytes from within the Activated Adult Heart after Injury. Nature.

[87] Van Wijk B., Gunst Q.D., Moorman A.F.M., Van Den Hoff M.J.B. (2012). Cardiac Regeneration from Activated Epicardium. PLoS ONE.

[88] Zhou B., Ma Q., Rajagopal S., Wu S.M., Domian I., Rivera-Feliciano J., Jiang D., Von Gise A., Ikeda S., Chien K.R. (2008). Epicardial Progenitors Contribute to the Cardiomyocyte Lineage in the Developing Heart. Nature.

[89] Wei K., Serpooshan V., Hurtado C., Diez-Cuñado M., Zhao M., Maruyama S., Zhu W., Fajardo G., Noseda M., Nakamura K. (2015). Epicardial FSTL1 Reconstitution Regenerates the Adult Mammalian Heart. Nature.

[90] McCarty G., Awad O., Loeb D.M. (2011). WT1 Protein Directly Regulates Expression of Vascular Endothelial Growth Factor and Is a Mediator of Tumor Response to Hypoxia. J. Biol. Chem..

[91] McCarty G., Loeb D.M. (2015). Hypoxia-Sensitive Epigenetic Regulation of an Antisense-Oriented lncRNA Controls WT1 Expression in Myeloid Leukemia Cells. PLoS ONE.

[92] Wagner K.-D., Wagner N., Wellmann S., Schley G., Bondke A., Theres H., Scholz H. (2003). Oxygen-regulated Expression of the Wilms’ Tumor Suppressor *Wt1* Involves Hypoxia-inducible Factor-1 (HIF-1). FASEB J..

[93] Von Gise A., Zhou B., Honor L.B., Ma Q., Petryk A., Pu W.T. (2011). WT1 Regulates Epicardial Epithelial to Mesenchymal Transition through β-Catenin and Retinoic Acid Signaling Pathways. Dev. Biol..

[94] Bax N.A.M., Oorschot A.A.M., Maas S., Braun J., Tuyn J., Vries A.A.F., Groot A.C.G., Goumans M.-J. (2011). In Vitro Epithelial-to-Mesenchymal Transformation in Human Adult Epicardial Cells Is Regulated by TGFβ-Signaling and WT1. Basic Res. Cardiol..

[95] Li N., Rignault-Clerc S., Bielmann C., Bon-Mathier A.-C., Déglise T., Carboni A., Ducrest M., Rosenblatt-Velin N. (2020). Increasing Heart Vascularisation after Myocardial Infarction Using Brain Natriuretic Peptide Stimulation of Endothelial and WT1+ Epicardial Cells. eLife.

[96] Wagner K.-D., Cherfils-Vicini J., Hosen N., Hohenstein P., Gilson E., Hastie N.D., Michiels J.-F., Wagner N. (2014). The Wilms’ Tumour Suppressor Wt1 Is a Major Regulator of Tumour Angiogenesis and Progression. Nat. Commun..

[97] Limana F., Bertolami C., Mangoni A., Di Carlo A., Avitabile D., Mocini D., Iannelli P., De Mori R., Marchetti C., Pozzoli O. (2010). Myocardial Infarction Induces Embryonic Reprogramming of Epicardial C-Kit+ Cells: Role of the Pericardial Fluid. J. Mol. Cell. Cardiol..

[98] Rudat C., Kispert A. (2012). Wt1 and Epicardial Fate Mapping. Circ. Res..

[99] Wagner N., Wagner K.-D. (2021). Every Beat You Take—The Wilms′ Tumor Suppressor WT1 and the Heart. Int. J. Mol. Sci..

[100] Godwin J.W., Debuque R., Salimova E., Rosenthal N.A. (2017). Heart Regeneration in the Salamander Relies on Macrophage-Mediated Control of Fibroblast Activation and the Extracellular Landscape. npj Regen. Med..

[101] Lepilina A., Coon A.N., Kikuchi K., Holdway J.E., Roberts R.W., Burns C.G., Poss K.D. (2006). A Dynamic Epicardial Injury Response Supports Progenitor Cell Activity during Zebrafish Heart Regeneration. Cell.

[102] Sallin P., De Preux Charles A.-S., Duruz V., Pfefferli C., Jaźwińska A. (2015). A Dual Epimorphic and Compensatory Mode of Heart Regeneration in Zebrafish. Dev. Biol..

