# Supplementary material for: Spiny Mice Show a Profibrotic Epicardial Mesothelial Response to Hypoxic Injury Comparable to C57BL/6 Mice
Source: Biomolecules. 2026 May 13;16(5):717. doi: 10.3390/biom16050717 (PMC13204827; doi:10.3390/biom16050717)
Supplement: Supplementary file 1 [file biomolecules-16-00717-s001.zip › biomolecules-4250136-supplementary.pdf]

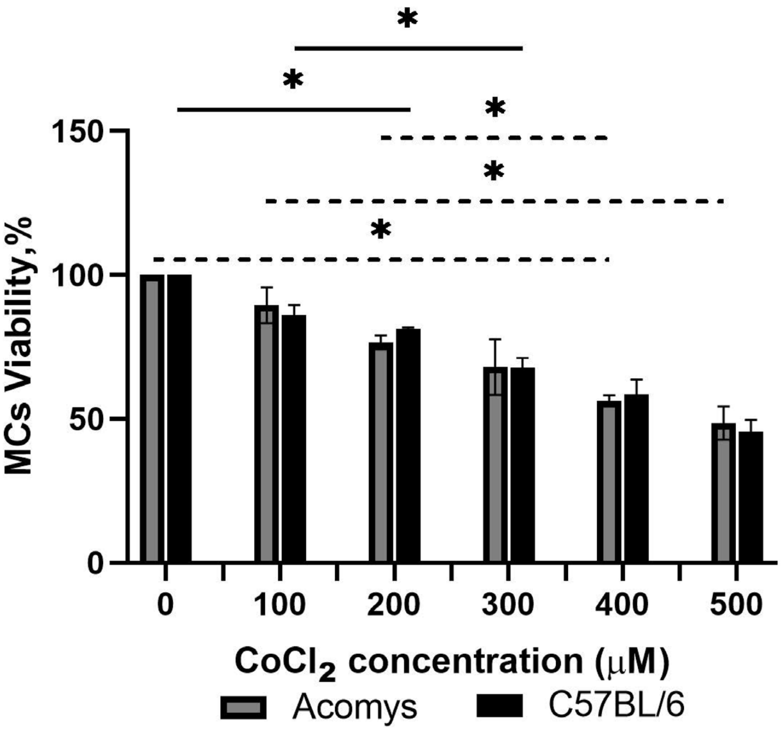

Figure S1. Graph showing quantitative assessment of survival of cardiac MCs from Acomys and C57BL/6 mice after $CoCl_2$ treatment.

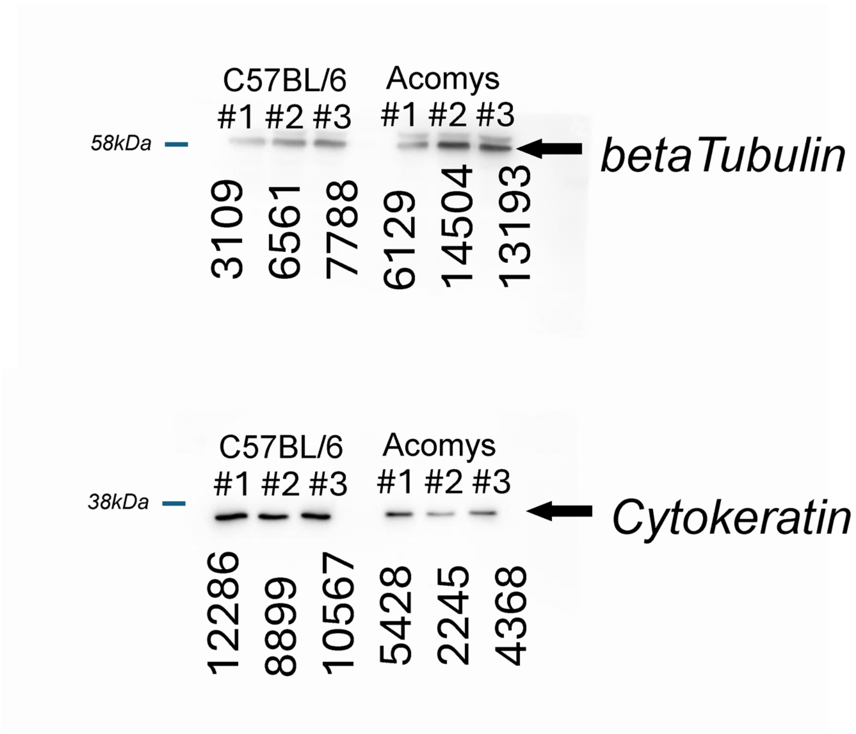

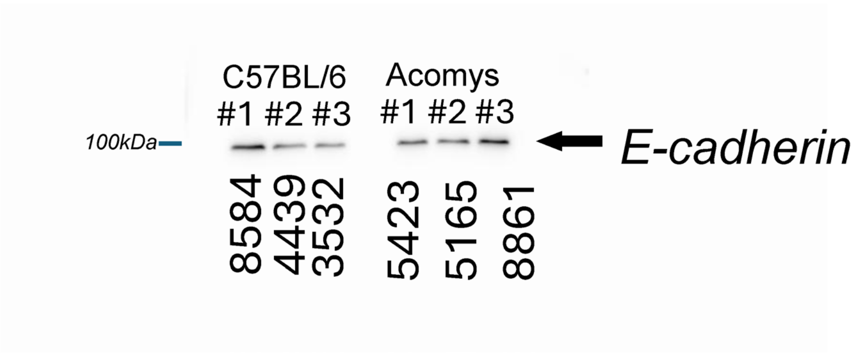

Figure S2. Original immunoblotting images showing the expression of E-cadherin, cytokeratin, and tubulin proteins in MCs obtained from the hearts of Acomys cahirinus and C57BL/6 mice.

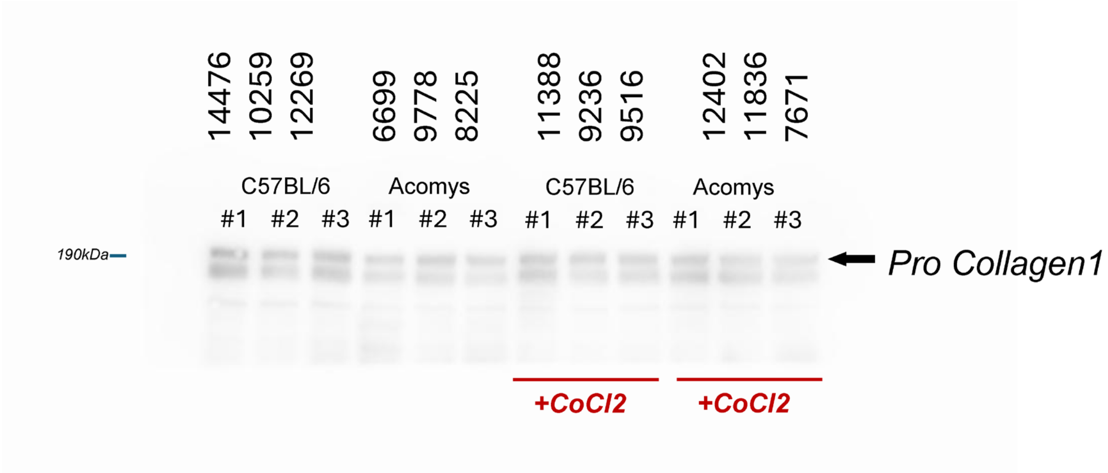

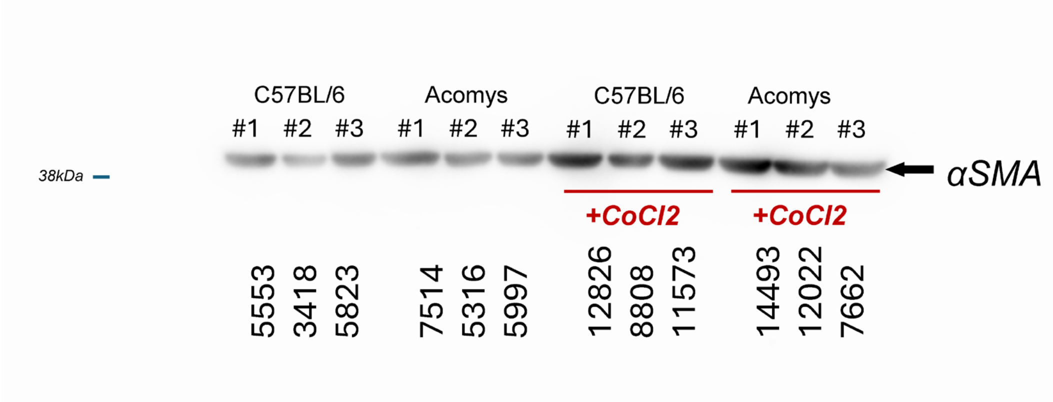

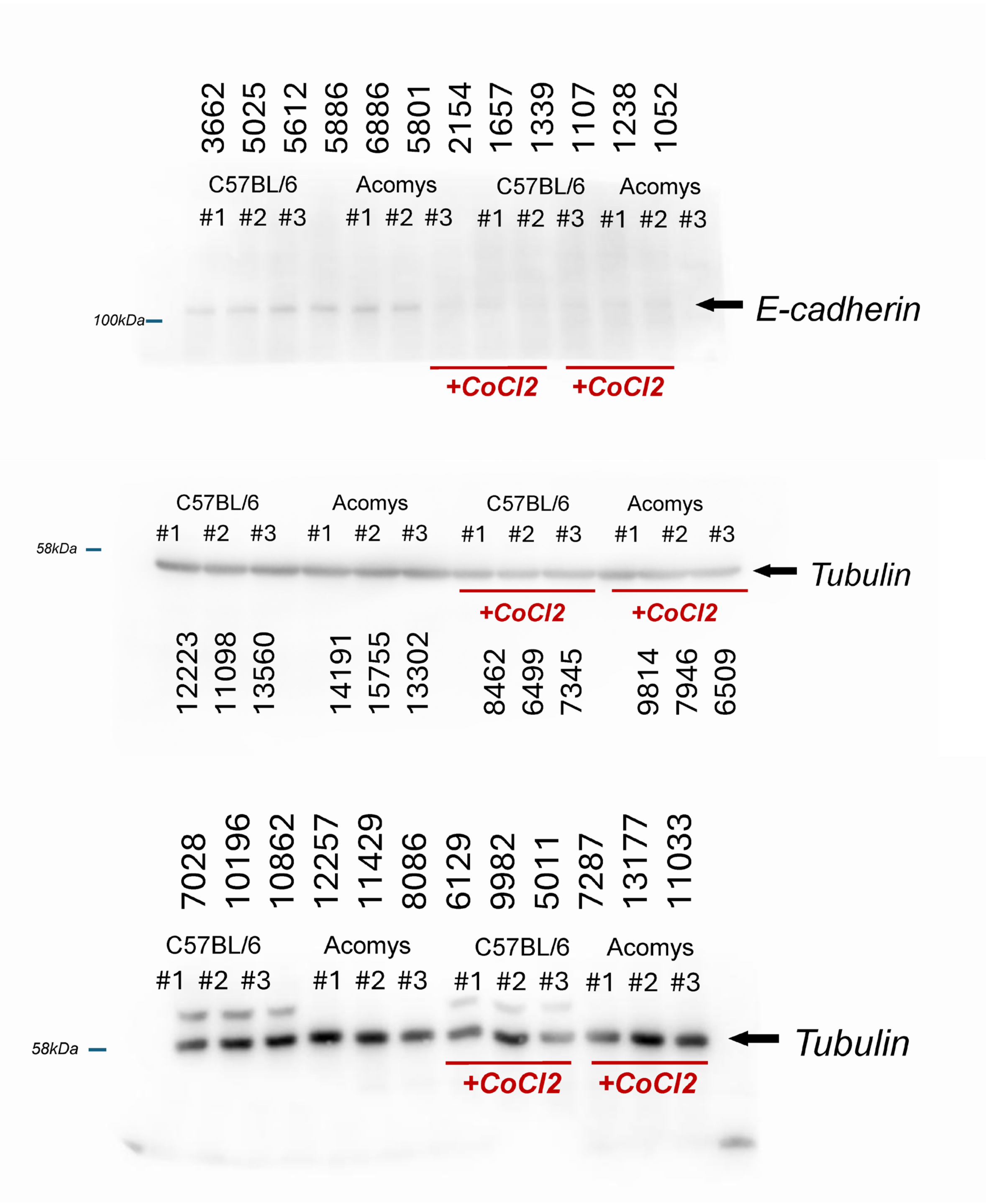

Figure S3. Original immunoblotting images showing the expression of E-cadherin, collagen I, α-SMA, and tubulin proteins in MCs obtained from the hearts of Acomys cahirinus and C57BL/6 mice under control conditions and after $CoCl_2$ treatment.
